# Supplementary material for: Self-assembly of nanocrystal checkerboard patterns via non-specific interactions
Source: Nat Commun. 2024 May 9;15:3913. doi: 10.1038/s41467-024-47572-2 (PMC11081958; doi:10.1038/s41467-024-47572-2)
Supplement: Supplementary file 1 — Supplementary Information [file 41467_2024_47572_MOESM1_ESM.pdf]

Supplementary Information:

Self-assembly of nanocrystal checkerboard patterns via non-specific interactions

Authors:

Yufei Wang[1,2†], Yilong Zhou[3†], Quanpeng Yang[3], Rourav Basak[4], Yu Xie[1], Dong Le[4], Alexander Fuqua[1], Wade Shipley[1,2], Zachary Yam[1], Alex Frano[4], Gaurav Arya[3*], and Andrea R. Tao[1,2‡]

Affiliations:

[1]Department of Chemical and NanoEngineering, University of California San Diego, La Jolla, CA 92023-0448 USA

[2]Materials Science and Engineering Program, University of California San Diego, La Jolla, CA 92023 USA

[3]Department of Mechanical Engineering and Materials Science, Duke University, Durham, NC 27708 USA

[4]Department of Physics, University of California San Diego, La Jolla, CA 92093 USA

[*] Email: gaurav.arya@duke.edu

[‡] Email: atao@ucsd.edu

† These authors contributed equally

Supplementary Note 1: Calculation of effective ligand shell thickness

Ligand shell thickness (L) was estimated using the following equations:

$$V = l^3 = \frac{4}{3}\pi r_\mathrm{h}^3 \, , \tag{1}$$

$$L = \frac{D_\mathrm{h}}{2} - r_\mathrm{h} \, , \tag{2}$$

where l is the edge length of a single cube analyzed using ImageJ software, r_h is the effective radii calculated using Eq. (1), D_h is hydrodynamic diameter measured from diameter distribution by volume from dynamic light scattering (DLS).

Supplementary Note 2: Calculation of surface thiol density

Assuming a set of cubic AgNCs saturated with thiol ligands on the surface, the average number of Ag atoms per AgNC ($N_{\mathrm{Ag/AgNC}}$) is:

$$N_{\mathrm{Ag/AgNC}} = \frac{l^3 \rho_{\mathrm{Ag}}}{M_{\mathrm{Ag}}} \, , \tag{3}$$

and the number of sulfur atoms per AgNC($N_{\mathrm{S/AgNC}}$) is:

$$N_{\mathrm{S/AgNC}} = k \cdot 6l^2 \, , \tag{4}$$

where l is the average edge length of AgNC, ρ_{Ag} is the density of silver, M_{Ag} is the atomic weight of silver and k is the surface ligand density of thiol. By combining eq.3 and eq.4, the Ag/S ratio can be represented as:

$$\frac{N_{\mathrm{Ag/AgNC}}}{N_{\mathrm{S/AgNC}}} = \frac{\frac{l^3 \rho_{\mathrm{Ag}}}{M_{\mathrm{Ag}}}}{k \cdot 6l^2} = 9.757 \cdot l \cdot k^{-1} \, , \tag{5}$$

where silver and sulfur concentrations and their ratio can be determined using ICP-MS.

Supplementary Note 3: SEM image analysis

Here we employ the method of angular cross-correlation analysis in reciprocal space to identify and compare the strength of different local orders in a globally disordered system in real space. The flowchart for the method is described in Supplementary Fig. 1. The method begins with collecting scanning electron microscopy images of the assembled nanoparticles keeping the field of view the same for every image. Then, each image is binarized and cropped to 1000 × 1000 pixels: amounting to an area of 4.5 × 4.5 $\mu\mathrm{m}^2$. A subsequent two-dimensional Fourier transformation is taken on these images to simulate a coherent small angle scattering pattern around the momentum transfer vector, (0,0).

Next, we evaluate Angular Cross-Correlation (ACC) of the intensity distribution defined through the cross-correlation function as Equation (6):

$$C_q(\Delta) = \frac{\langle I(q,\phi) I(q,\phi+\Delta)\rangle_\phi - \langle I(q,\phi)\rangle_\phi^2}{\langle I(q,\phi)\rangle_\phi^2} \tag{6}$$

Here, q denotes the radius at which we evaluate the ACC. While φ is the angular coordinate of a speckle, Δ is a measure of the angular separation between two points in reciprocal space as indicated in Fig. 1e. Angular brackets measure the average in the following sense: $\langle f \rangle_\phi = \frac{1}{2\pi}\int_0^{2\pi} f \, \mathrm{d}\phi$. ACC analysis on coherent small angle X-ray scattering had been previously applied to identify local orientational symmetry of single particles in a disordered assembly of those single particles, such as in colloids[1-3]. Here we utilize the method and expand on its

remarkable capability of picking out the strength of different local orders for finitely many scatterers. The ACC is then Fourier-transformed into inverse-angle space to yield the nth Fourier coefficient C_q^n, then plotted as a function of Fourier index n (Fig. 1f).

$$C_q^n = \frac{1}{\pi} \int_0^{\pi} C_q(\Delta) \cos(n\Delta)\, \mathrm{d}\Delta \tag{7}$$

The q-value is determined based on the interparticle connection of the local order being investigated. At that q, the value of C_q^n corresponds to the strength of orientational symmetry. For example, to identify the symmetry of the checkerboard pattern we calculate ACC at $q = \pi a^{-1}$, where a is the side length of each nano cube and find the strength of 4-fold symmetry (i.e., n=4). Fig. 1F highlights this connection to local order by showing a pronounced strength of 4-fold symmetry from a simulated checkerboard pattern. Now, following this analysis we can see that the "PEG:C16 = 50 µM:6 µM" sample reveals considerable amount of local checkerboard order even though there is no global order present. This is in contrast with the "all PEG" sample which doesn't show any local order. For most of the morphology investigated, the nano cubes either form an edge-edge connection, or a face-face connection. For edge-edge connection $q = \pi a^{-1}$ was used, while majorly face-face connection contribution was seen at $q = \frac{2}{3}\pi a^{-1}$. Then for 1D connection we probe the 2-fold symmetry (n = 2) whereas for 2D connection, like the checkerboard, we investigate the 4-fold symmetry (n = 4) of the orientational order. From the SEM images of Fig. 3A we perform ACC analysis to find C_q^n plotted in Fig. 3b to find and compare the local order. The entire ACC analysis is presented in Supplementary Fig. 2.

Supplementary Note 4: Iterative feedback between coarse-grained molecular dynamics (CG-MD) simulations

The phrase "iterative feedback" refers generically to the feedback loop where experimentally determined parameters (e.g., polymer graft length, chemistry, and density) are used to implement computational models and, in turn, simulation results are used to provide design feedback to the experiments. In the specific context of generating checkerboard lattices, we started by synthesizing nanocube building blocks within only a small phase space (e.g., number percentage of hydrophobic ligands = 75%). We next used CG-MD simulations to model nanocube self-assembly over a larger phase space to identify the critical influence of parameters such as ligand graft density and ligand percentage. We then carried out assembly experiments to validate the simulation results. If the experimental results agree with the simulations, we then use simulations to identify the experimental parameters required for checkerboard assembly. This process was repeated until we were able to develop a protocol identifying the experimental parameters (e.g., ligand exchange solvent and feedstock concentrations) for checkerboard assembly.

Supplementary Note 5: UV-Vis-NIR Reflection Measurements

After drop casting Ag NCs on the air-water interface, reflection UV-Vis-NIR measurements were obtained by illuminating the Ag NC film using a tungsten-halogen lamp (≈10 mW, ThorLabs). The cross-sectional area of the broadband white light sources was measured at 8.6 mm^2. The reflected signal was captured through a fiber optic reflection probe and measured by CCD spectrometer (ThorLabs). Reflectance spectra were obtained by averaging 100 acquisitions with an acquisition time of 20-40 ms as needed to maximize signal without oversaturating the detector.

Supplementary Note 6: Optical Simulations

Finite-difference time-domain (FDTD) simulations were performed using the 3D Electromagnetic Simulator FDTD Solver 2023 R1 from Lumerical Solutions, Inc. The simulations were run on an Intel i7 2.1GHz Processor with 64 GB of RAM. Optical constants for Ag and water were taken from Palik[4]. The nanocubes are floating in water with only the top surface exposed to air with a background refractive index of n=1.

The Ag NCs have an 80 nm edge length and a 10 nm edge radius. To simulate 2D lattices of NCs, a periodic boundary was used. To emulate dispersed NCs, the NCs were modelled with a gap distance of 100 nm which results in negligible plasmonic coupling. To simulate edge-edge and checkerboard assembled structures, the NCs were placed in an infinite 2D checkerboard pattern with a gap distance of 4 nm edges of adjacent nanocubes. A plane wave source was used to illuminate the nanocubes in the negative z direction. A power monitor above the source was used to capture reflection spectra. The light source has a magnitude of 1 V m^{-1} with a broadband irradiation wavelength range of 300-1000 nm.

Supplementary Note 7: Details of Phase Diagram

The phase diagram (Fig. 2b) shows the emergence of a total of six distinct phases, which were qualitatively categorized as dispersed, 1D, or 2D (i.e., face-face 2D, checkerboard, face-face 1 D, edge-edge 2D, edge-edge 1D, and dispersed).

Briefly, when the solvent is water, the effective interactions between hydrophilic ligands are repulsive while those between hydrophobic ligands are attractive. Therefore, attraction between NCs would increase with respect to the increasing percentage of hydrophobic ligands, which leads to the phase transition from dispersed state (where hydrophilic ligands dominate) to 1D (where the interplay of the two types of ligands allows NCs to assemble along one lateral direction), and to 2D (where hydrophobic ligands dominate). Interestingly, the interplay of the hydrophilic and the hydrophobic ligands can also give rise to different connections (edge-edge or face-face) between NCs. The free energy profiles $\Delta F(d)$ computed for edge-edge and face-face contacts show that face-face contacts emerge at higher percentage of hydrophobic ligands (Supplementary Fig.14). This is because the longer hydrophilic ligands tend to stretch away from the NC surface and to prevent NCs from forming face-face contacts until the NC surfaces are dominantly covered by hydrophobic ligands (high percentage of hydrophobic ligands). Related to this, the assembly of NCs emerge at larger percentage of hydrophobic ligands when it comes to higher grafting density. This is because the ligands tend to stretch more with the increase in the overall grafting density, leading to stronger repulsion between NCs. As a result, NCs assemble into various structural phases including dispersed phase, 1D strings with either edge-edge or face-face contacts, and 2D structures with face-face contacts (Fig. 2b).

In addition, orientations of NCs can also play an important role in their assemblies. Based on the occluded area theory, NCs trapped at interfaces would adopt certain orientations to maximize the occluded areas with the interface. Given that the hydrophilic ligands tend to pull NCs down to the water layer and the hydrophobic ligands do the reverse, the equilibrium positions of NCs at the interface are affected by the percentage of the two types of ligands. Not only the NC cores can occlude interfacial areas, but the stretching ligands also do (the more stretching the ligands have, the more interfacial area they occlude). As a result, the orientations of NCs are determined by both the grafting density and the percentage of hydrophobic ligands. Interestingly, at large percentage of hydrophobic ligands (> 67%), face-up oriented NCs mainly assemble into 2D structures with face-face contacts while edge-up oriented NCs form 1D strings with face-face contacts and vertex-up oriented NCs form 2D structures with edge-edge

contacts. The edge-up oriented NCs can maximize their interparticle interactions through the face-face connections without altering their favorable orientations, resulting in the formation of 1D strings with edge-up oriented NCs. Even though face-face contacts could gain more interaction energies than the edge-edge contacts, vertex-up oriented NCs would have to sacrifice more interfacial energies by rotating themselves at the interface to fit in the face-face contacts, making 2D structures with edge-edge contacts most favorable.

In summary, both interparticle interactions and orientations of NCs play significant roles in the assembled structural phases, where the interparticle interactions are affected by the interplay of the two types of ligands and the orientation of such grafted NCs are determined by many factors including interfacial displacement, occluded interfacial area, and chain stretching at the interface.

Supplementary Table 1 │ ICP-MS results for AgNCs functionalized with PEG20k and varied concentration of C_{16} in feedstock.

Molar ratio in feedstock, PEG:C_{16}	Ag/S ratio	Ligand density (ligands nm^{-2})	Phase from SEM
50µM:0 µM	1305.91	0.523	Dispersed
50µM:1 µM	417.48	1.636	Edge-edge 1D
50µM:3 µM	505.54	1.351	Checkerboard
50µM:6 µM	596.50	1.145	Checkerboard
50µM:9 µM	556.18	1.228	Checkerboard
50µM:9 µM	598.59	1.141	Face-face 1D
50µM:15 µM	826.86	0.826	Face-face 1D

Supplementary Table 2 | Comparison of hydrodynamic diameter ($D_{h,}$ in nm) of AgNCs (l = 88 ± 5 nm), in H_2O with surface coated with PEG (50 µM in feedstock) of varying M_w, and PEG + C_{16} (50 µM:6 µM in feedstock).

	PEG6k	PEG10k	PEG20k	PEG30k	PEG54k
D_h of PEG coated AgNCs	150.2	159.7	163.0	188.7	187.0
D_h of PEG + C_{16} coated AgNCs	154.0	162.0	164.0	169.0	175.0
Ligand shell thickness (L)	22.4	26.4	27.4	29.9	32.9
PEG to core ratio (L/88)	0.255	0.300	0.311	0.340	0.375

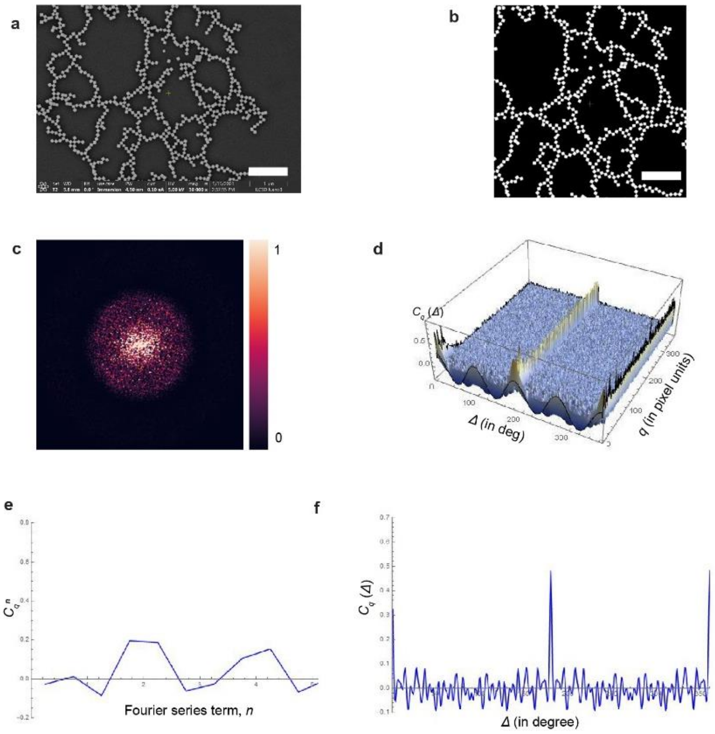

Supplementary Figure 1: Finding orientational symmetry in local structure from a globally disordered system —flowchart of steps. (a) Collected SEM image of the assembled nanoparticles Scale bar 1 μm. (b) Binarized and cropped SEM image into 1000×1000 pixels: amounting to an area of $4.5 \times 4.5\ \mu m^2$. Scale bar 1 μm. (c) Subsequent two-dimensional Fourier transformation taken on images in (b). (d-e) Angular Cross-Correlation (ACC) of the intensity distribution defined through using the cross-correlation function in Equation (6) and calculated ACC at $q = \pi a^{-1}$, where a is the side length of each nano cube and find the strength of 4-fold symmetry of the checkerboard pattern. (f) Fourier-transformed ACC into inverse-angle space to yield the nth Fourier coefficient C_q^n, then plotted as a function of Fourier index n using Equation (7). Source data are provided as a Source Data file.

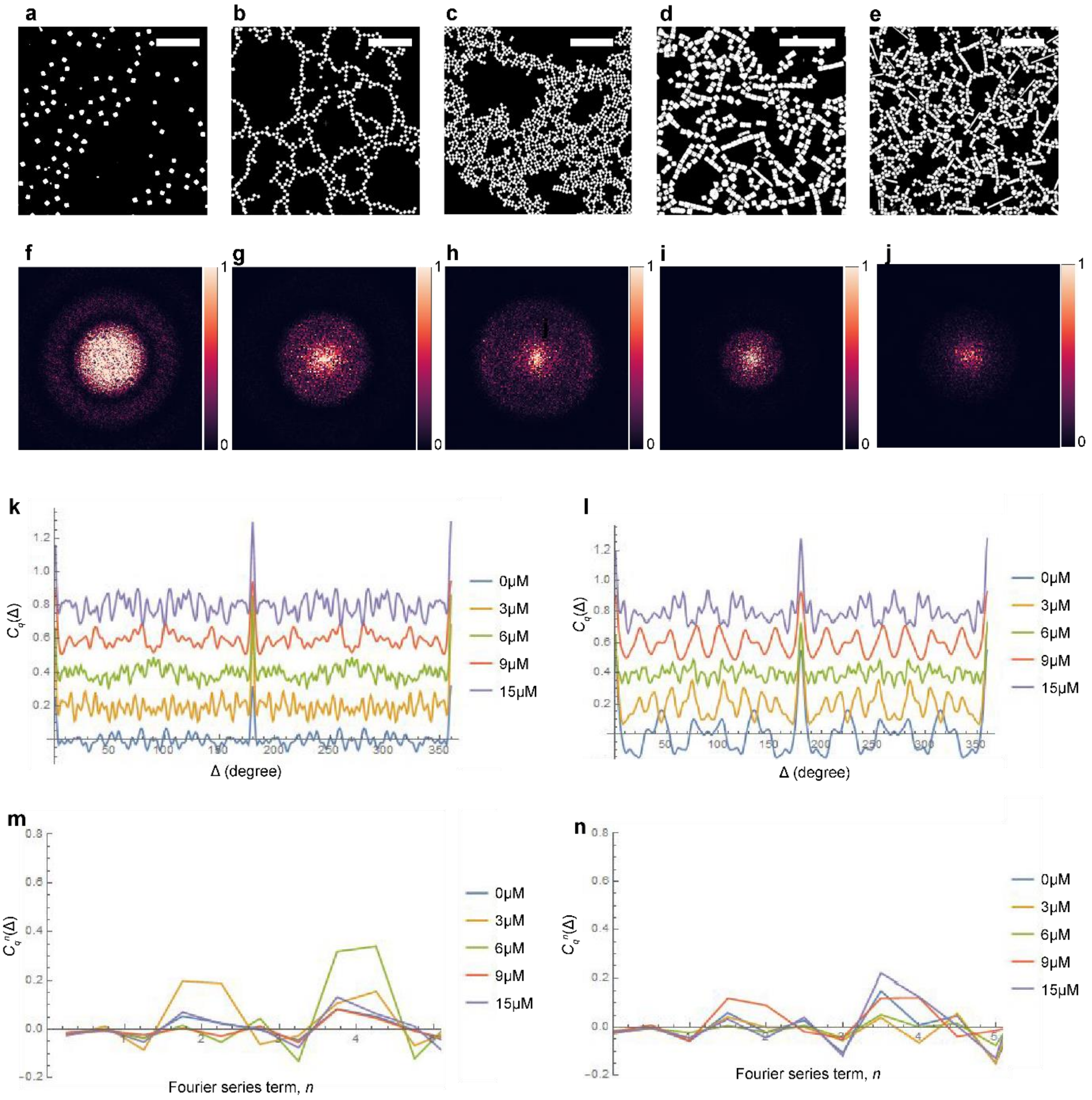

Supplementary Figure 2. Details about the evaluation of Angular Cross-correlation analysis and extraction of strength of n-fold symmetry from the cross-correlation function (a-e) SEM images of the assembly results using Ag NCs post-synthetically modified with the following feedstock concentrations: a) 0 µM b) 3 µM c) 6 µM d) 9 µM e) 15 µM C_{16} and 50 µM PEG20K. Scale bar = 1 µm (f-j) Amplitude of 2D Fourier transform of each SEM image in (a-e). (k-l) Cross correlation $q = \pi a^{-1}$ for k) and $q = \frac{2}{3}\pi a^{-1}$ for l). The lines are offset by an arbitrary for ease of visualization (m-n) Extraction of strength of n-fold orientational symmetry from $C_q(\Delta)$ with $q = \pi a^{-1}$ and (m) Extraction of strength of n-fold orientational symmetry from Cq(Δ) with $q = \frac{2}{3}\pi a^{-1}$ (n). Source data are provided as a Source Data file.

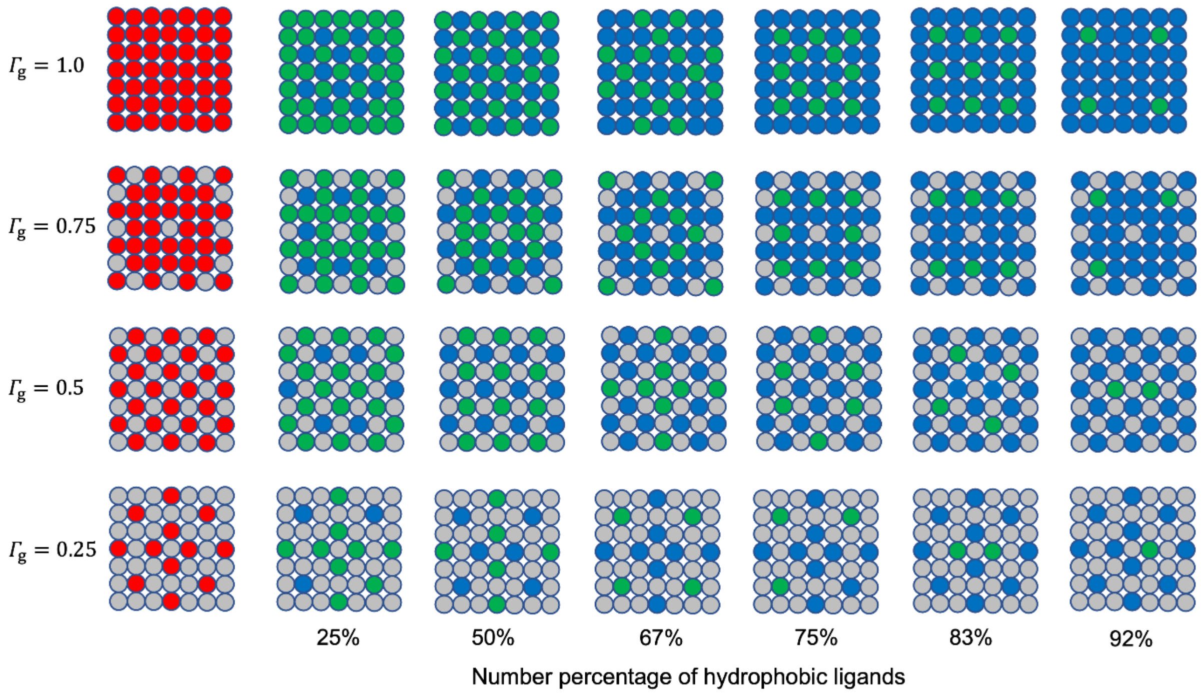

Supplementary Figure 3. Grafting patterns at each overall grafting densities (in units of chains σ^{-2}) and number percentage of hydrophobic ligands. The overall grafting sites at each grafting density are marked by red color. The grafting sites of hydrophilic and hydrophobic ligands are marked by green and blue colors, respectively.

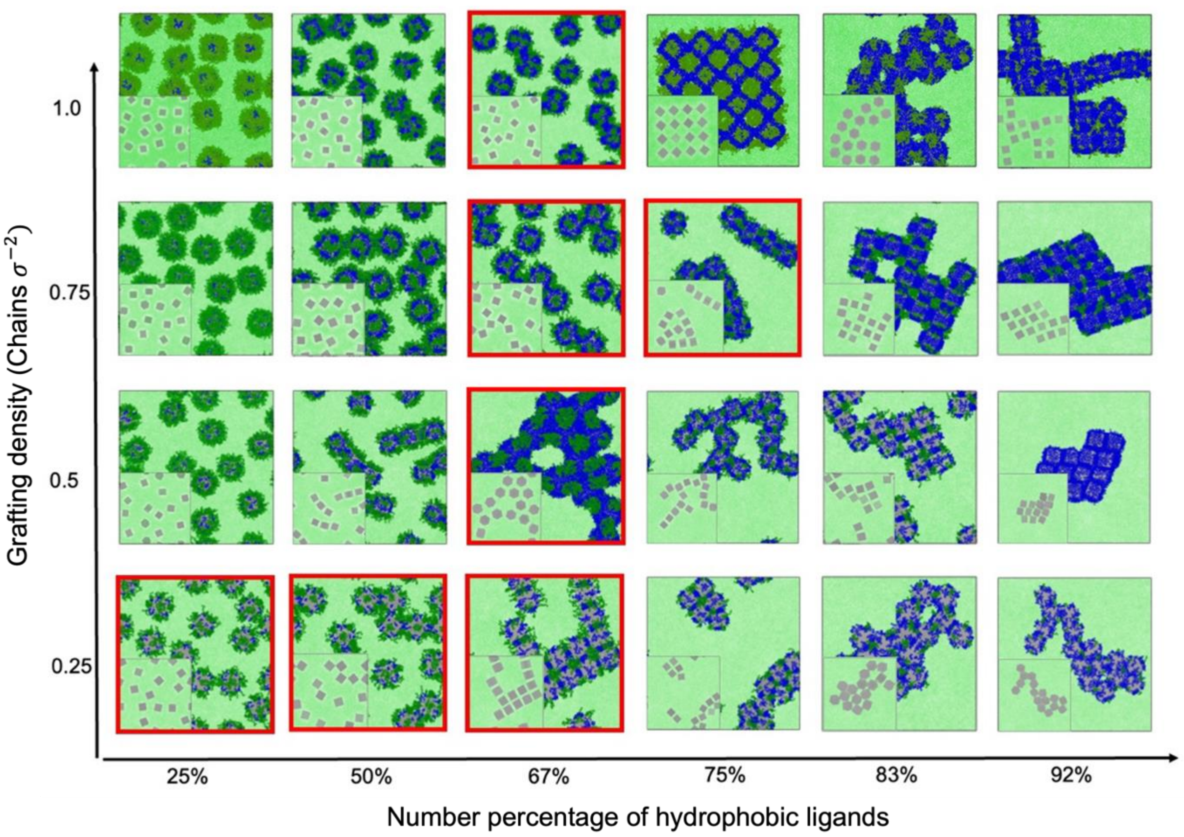

Supplementary Figure 4. Assembly results from CG-MD simulations of 16 grafted nanocubes with respect to grafting density and number percentage of hydrophobic ligands. Different phases coexisting in the conditions circled in red are shown in Supplementary Figs. 21-27.

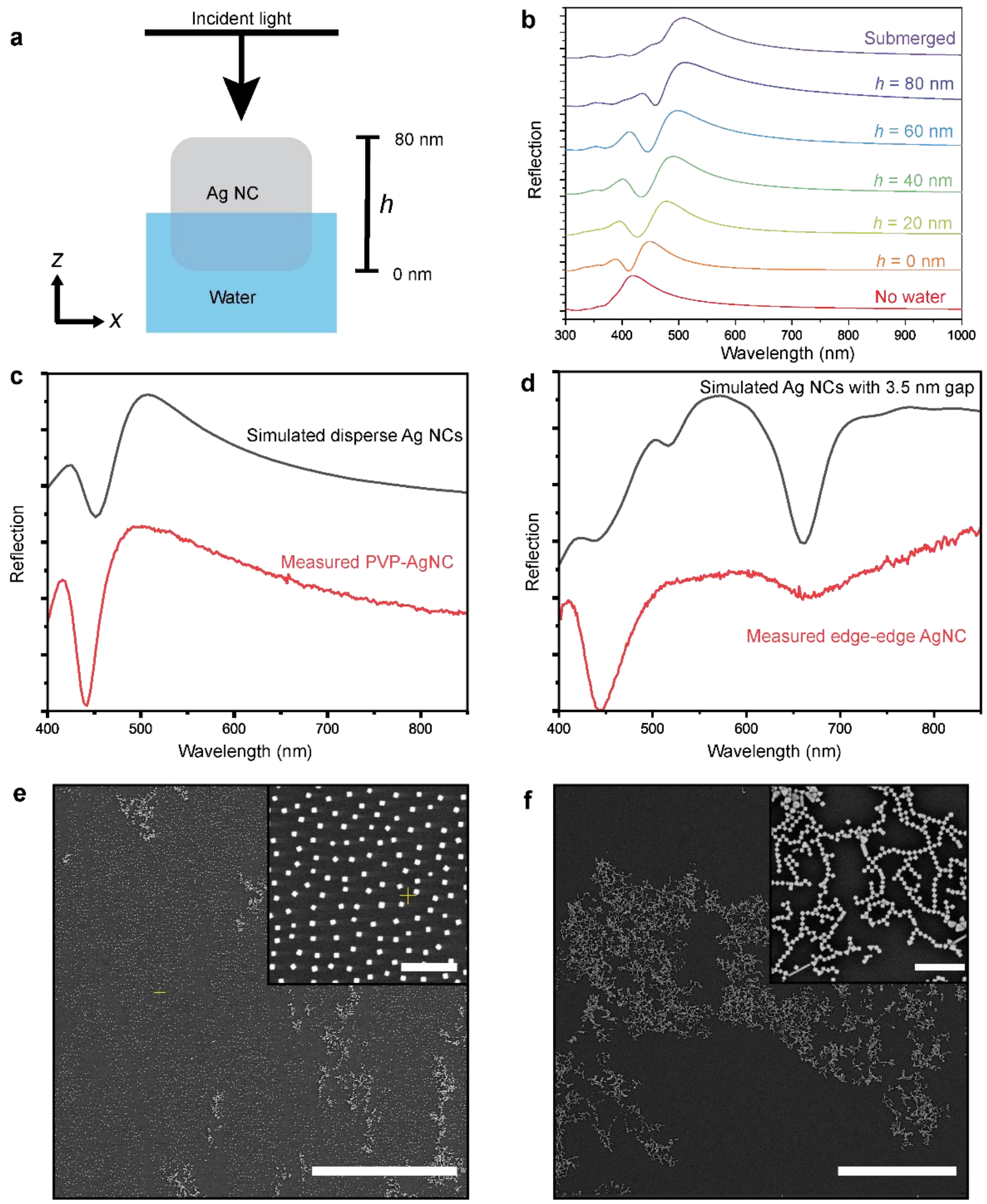

Supplementary Figure 5: FDTD simulations and UV-Vis-NIR reflection measurements of interfacial AgNC assemblies. (a) Diagram of FDTD model showing the varying height, *h*, of the air-water interface with the z-direction. (b) Simulated reflection spectra of a dispersed AgNC film with varying air-water interface locations as indicated by *h*. (c) Experimental (red line) and simulated (black line, *h*=70 nm) reflection spectra of dispersed well-separated PVP-AgNCs at the air-water interface. (d) Experimental (red line) and simulated (black line, 3.5 nm spacing) reflection spectra for edge-edge assembled AgNC at the air-water interface. (e) Corresponding SEM images of the dispersed PVP-AgNC film from panel C after transfer onto a solid Si substrate. (scale bar = 20 μm, inset scale bar = 1μm). (F) Corresponding SEM images of the edge-edge assembled AgNCs measured in panel D after transfer onto a solid Si substrate. (scale bar 20μm, inset 1μm).

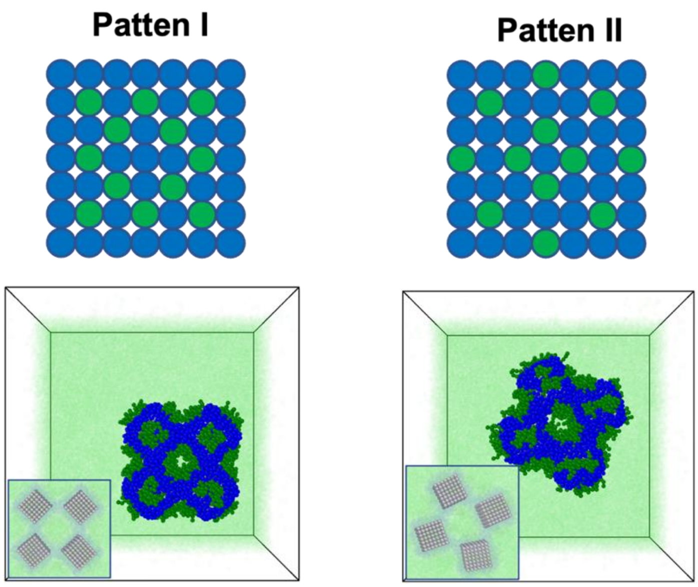

Supplementary Figure 6. Assembly results from CG-MD simulations of 4 grafted nanocubes with two different grafting patterns at $\Gamma_g = 1.0$ chains σ^{-2} and number percentage of hydrophobic ligands = 75% fixed.

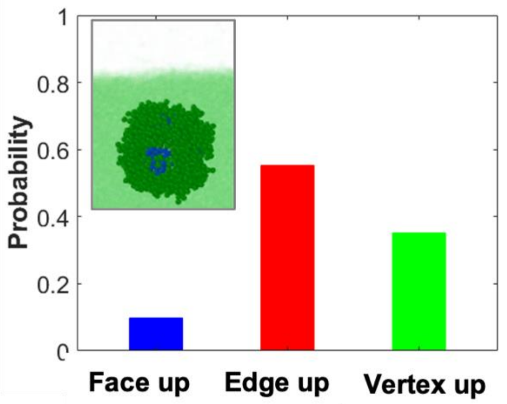

Supplementary Figure 7. Probability of a grafted nanocube exhibiting the three principal orientations obtained from a freely mobile nanocube with $\boldsymbol{\Gamma_g = 1.0}$ chains $\boldsymbol{\sigma^{-2}}$ and number percentage of hydrophobic ligands = 25% at the interface. Inset: Representative snapshot of the nanocube at the interface captured from the simulation. Source data are provided as a Source Data file.

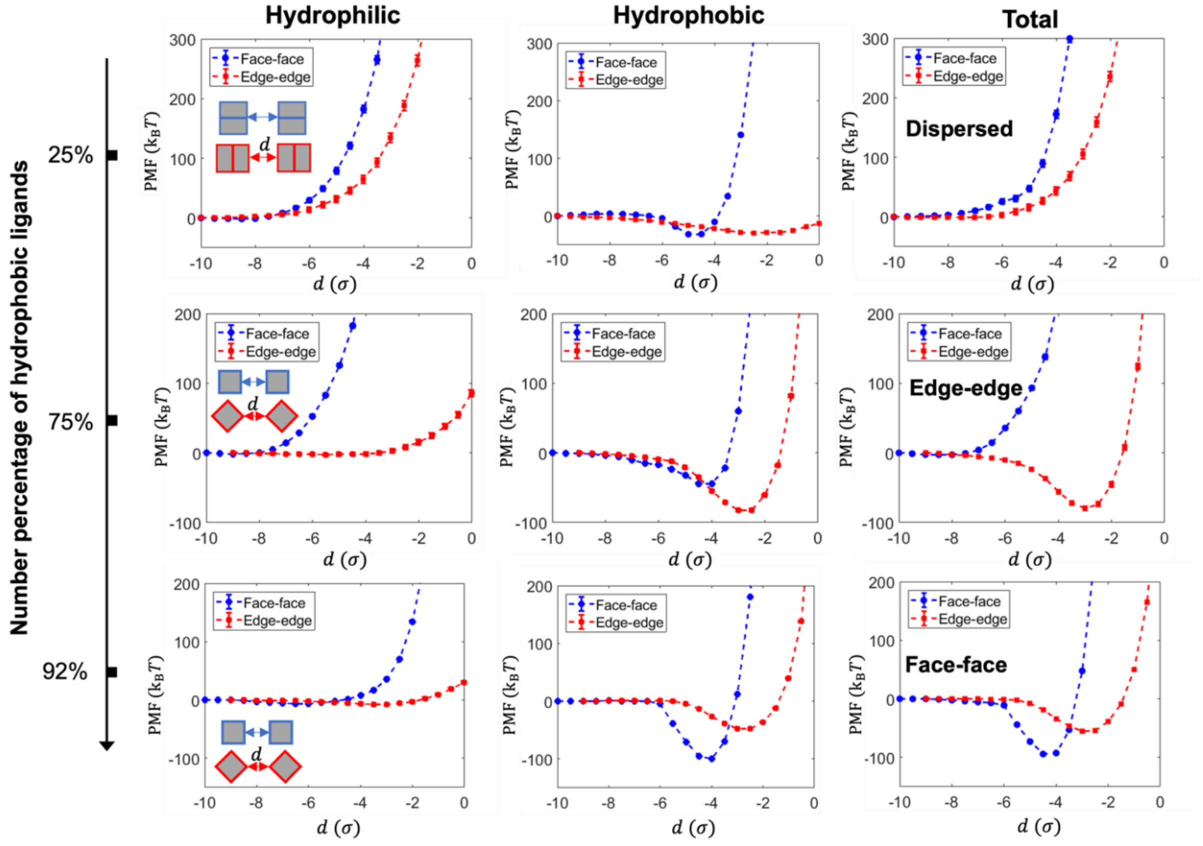

Supplementary Figure 8. Free energy of interaction (potential of mean force, PMF) between grafted NCs at $\boldsymbol{\Gamma_g = 1.0}$ chains $\boldsymbol{\sigma^{-2}}$ and its contributions from hydrophilic and hydrophobic grafts. Source data are provided as a Source Data file.

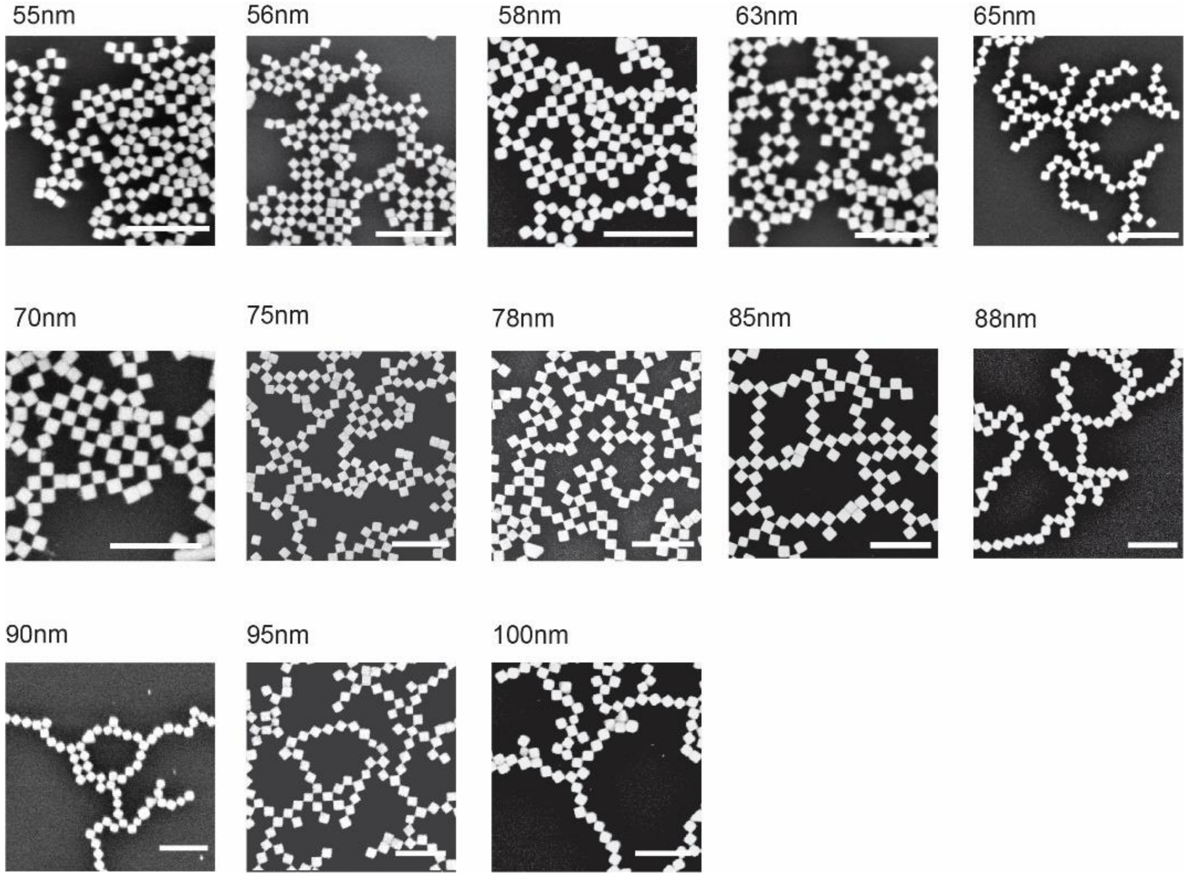

Supplementary Figure 9. Representative SEM images of assembled AgNCs of different sizes functionalized with 50 μM PEG20k and 6 μM C_{16} in feedstock (scale bar 1 μm).

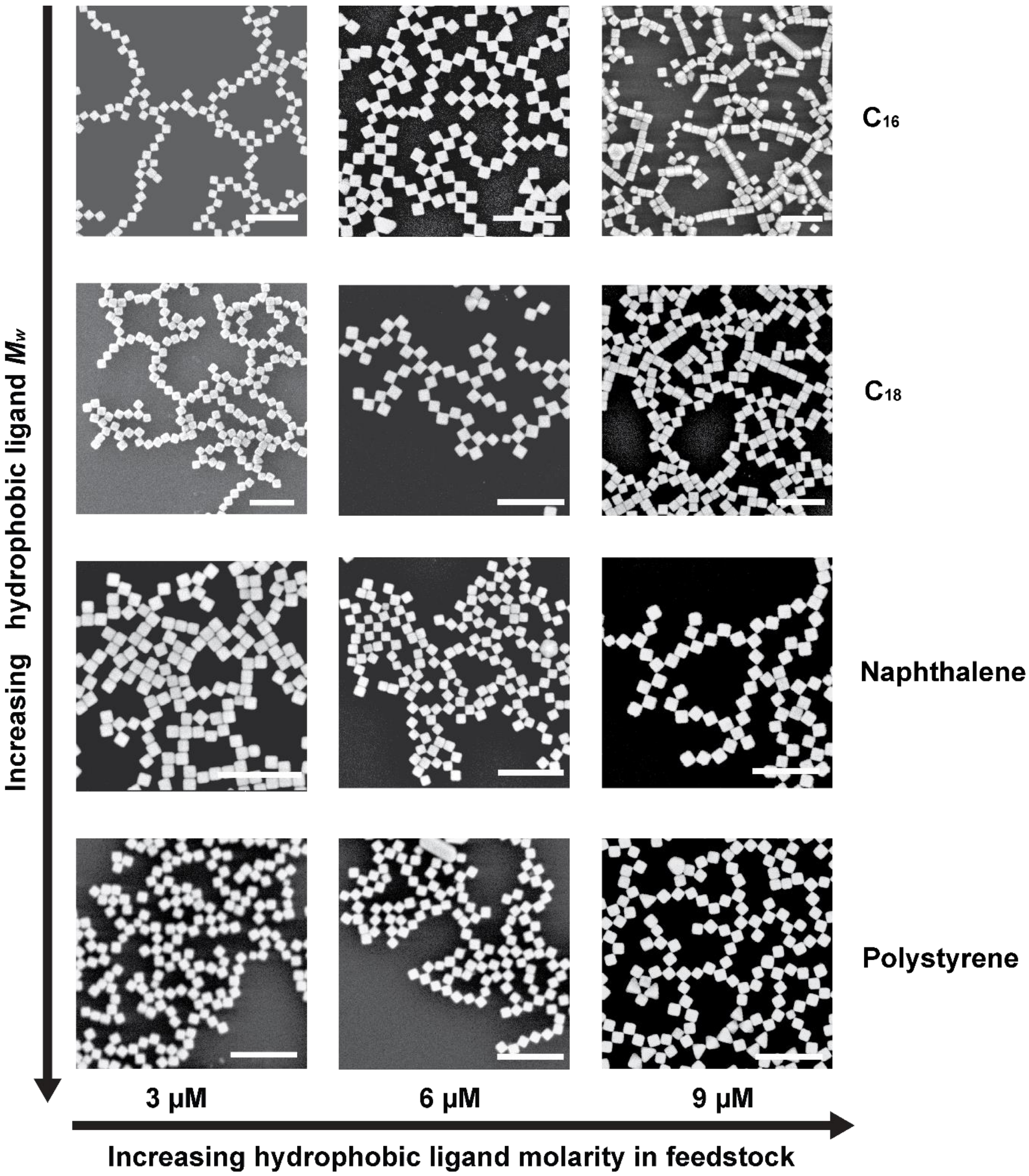

Supplementary Figure 10. Representative SEM images of assembled AgNCs functionalized with 50 µM PEG20k and different hydrophobic ligand with varied ligand ratio in feedstock (scale bar 500 nm).

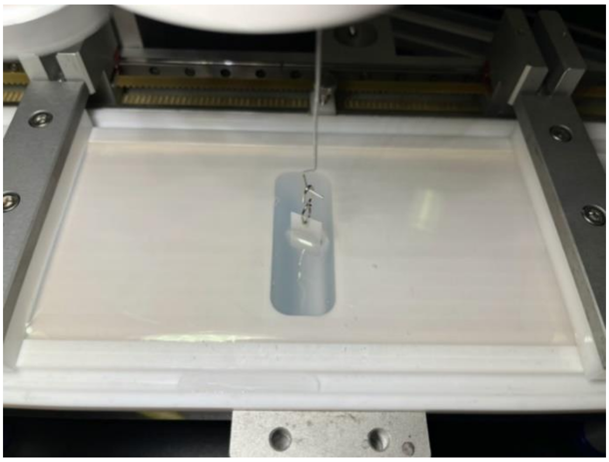

Supplementary Figure 11. Langmuir−Blodgett trough. Checkerboard assemblies can be captured from virtually everywhere on the air-water interface of the Langmuir−Blodgett trough (which is 36.5 cm × 7.5 cm in area).

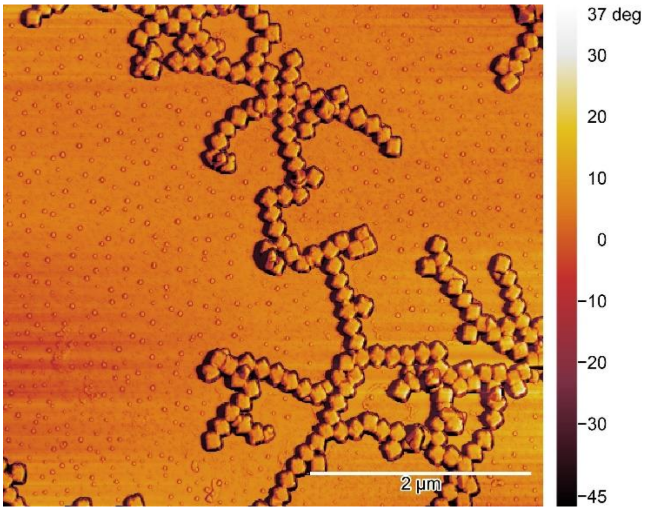

Supplementary Figure 12. AFM phase contrast image of edge-edge aligned Ag nanocubes. AFM phase contrast imaging indicates that there is no clustering of PEG ligands in the center of each facet and the ligand shell is homogeneous**.**

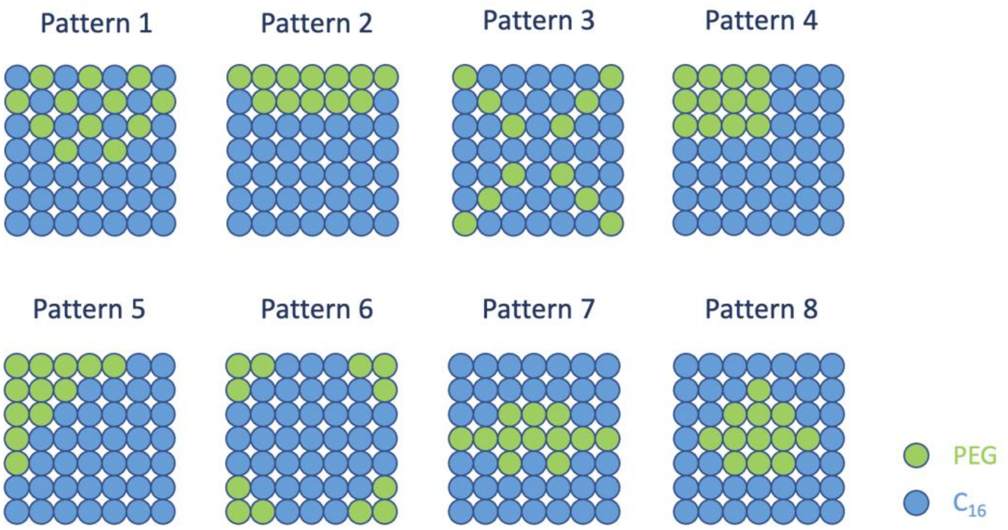

Supplementary Figure 13. Different grafting patterns explored in CG-MD simulations for the case of $\Gamma_g = 1.0$ chains σ^{-2} and number percentage of hydrophobic ligands = 75%, where checkerboard assembly was observed (also see Supplementary Figure 3). Most of the grafting patterns did not lead to checkerboard assembly; only Pattern 8, where the PEG chains are in the middle of the facet, still led to checkerboard formation.

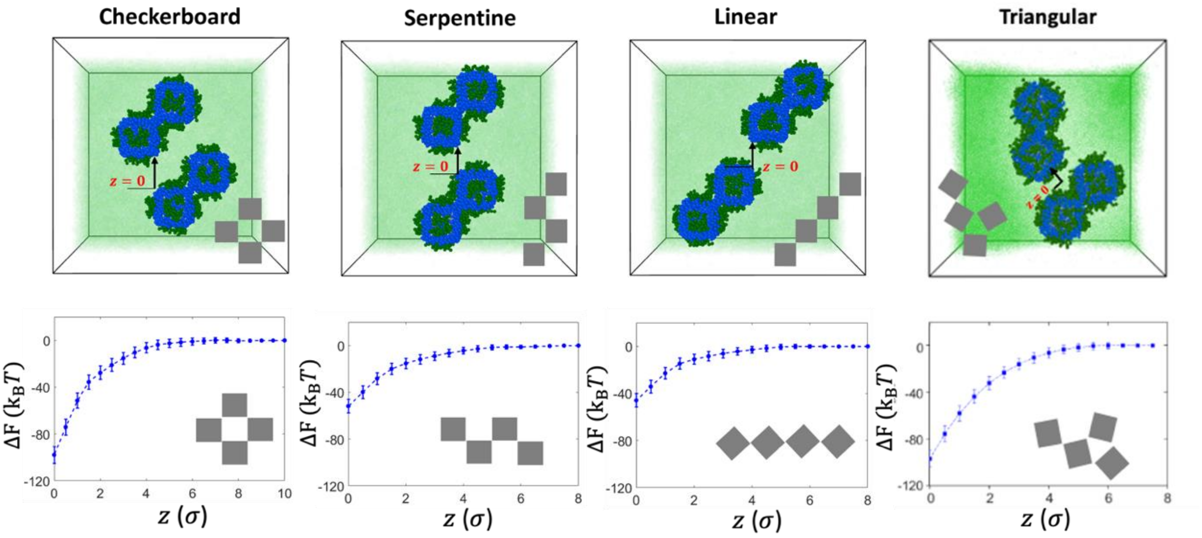

Supplementary Figure 14. Free energy profiles of four representative structures as a function of edge-edge distance between two NC dimers. The overall grafting density and percentage of hydrophobic ligands are 1.0 chains σ^{-2} and 75%, respectively, i.e., the checkerboard lattice formation condition in Fig. 2b. Calculations confirm that the checkerboard cluster and the triangle-shaped clusters exhibit the lowest free energy amongst similarly sized clusters (e.g., zigzag and linear). This suggests that the formation of triangular motifs is in fact almost as favorable as the formation of the checkerboard motif and that there is an energy barrier to overcome for rearranging a triangular cluster into a checkerboard cluster. Source data are provided as a Source Data file.

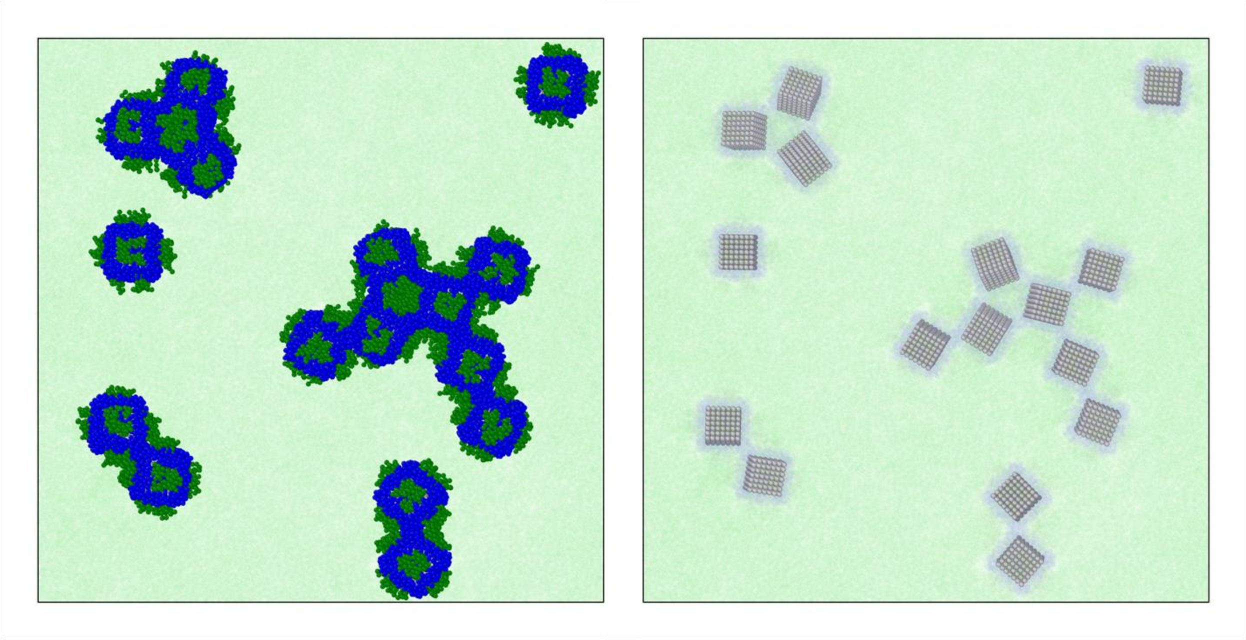

Supplementary Figure 15. Assembly results from CG-MD simulations of 16 grafted nanocubes with hydrophilic ligand length $l_{\mathrm{H_i}} = 6\sigma$, $\Gamma_{\mathrm{g}} = 1.0$ chains σ^{-2}, and number percentage of hydrophobic ligands = 75%.

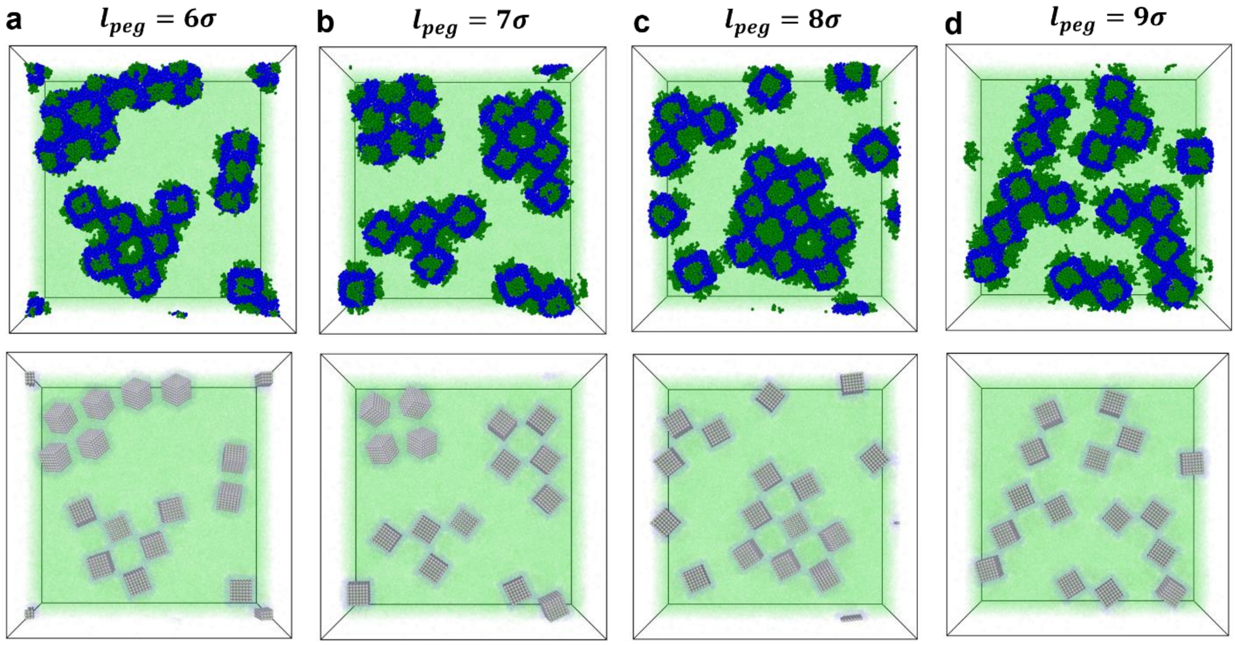

Supplementary Figure 16. Assembly results from CG-MD simulations of 16 grafted nanocubes at $\Gamma_{\mathrm{g}} = 1.0$ chains σ^{-2} and number percentage of hydrophobic ligands = 75% with respect to length of hydrophilic ligand length ligands. (a) $l_{\mathrm{H_i}} = 6\sigma$. (b) $l_{\mathrm{H_i}} = 7\sigma$. (c) $l_{\mathrm{H_i}} = 8\sigma$. (d) $l_{\mathrm{H_i}} = 9\sigma$. Bottom: nanocubes without grafts for a better visualization. Defects are marked by red circles.

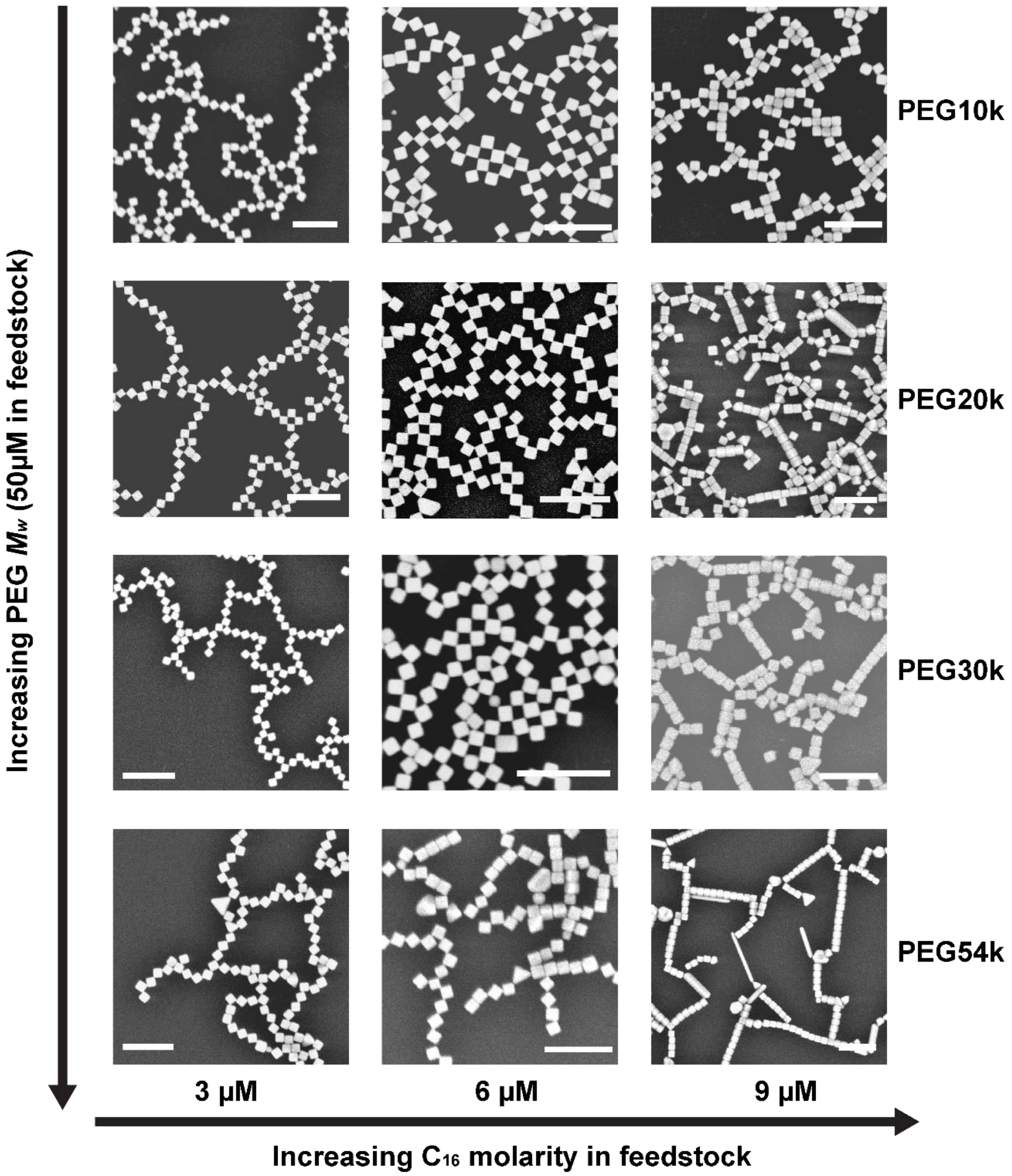

Supplementary Figure 17. Representative SEM images of assembled AgNCs functionalized with PEG of 10k-54k and C_{16} with varied ligand ratio in feedstock (scale bar 500 nm). Based on the assembly results, the AgNCs modified with C_{16} + PEG10k/20k/30k all show phase transform from 1D edge-edge to checkerboard and then to 1D face-face as the molarity of C_{16} in feedstock increases. The AgNCs functionalized with C_{16} + PEG54k directly transform from 1D edge-edge to 1D face-face as increasing the molarity of C_{16} in feedstock and no checkboard phase was observed.

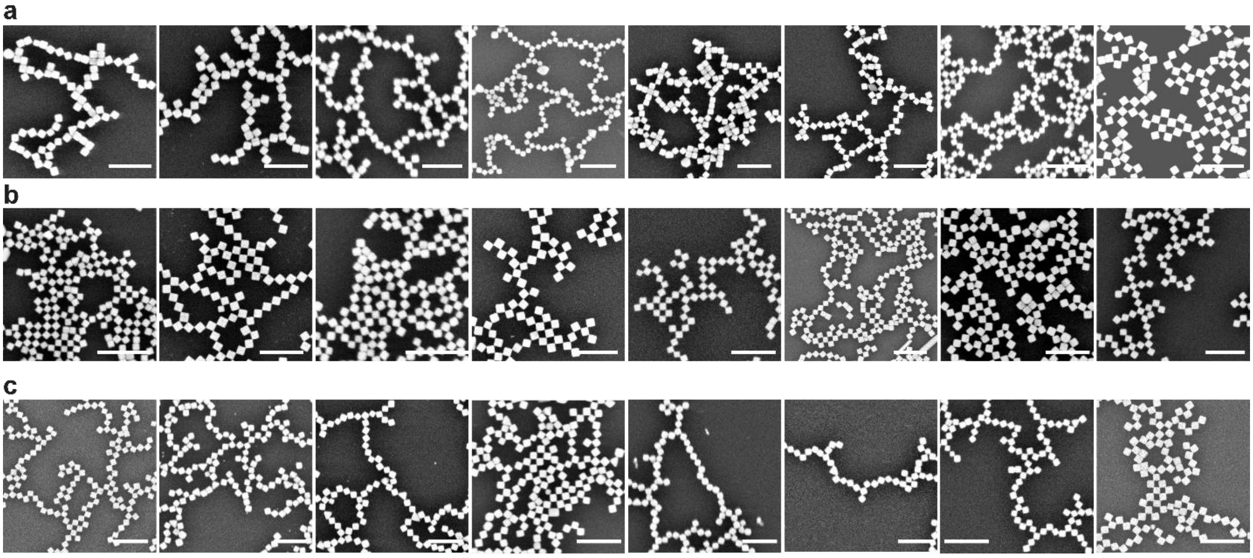

Supplementary Figure 18. Representative SEM images of assembled AgNCs functionalized with (a) PEG10k & C_{16}, (b) PEG20k & C_{16} (c) PEG30k & C_{16} with ligand ratio of 50µM:6 µM (PEG:C_{16}) in feedstock (scale bar 500 nm). Each image represents a different AgNC sample. Based on the assembly results, even though all three PEG length are able to lead to formation of checkerboard lattice, the PEG20k gives the most rigid and uniform checkerboard lattice.

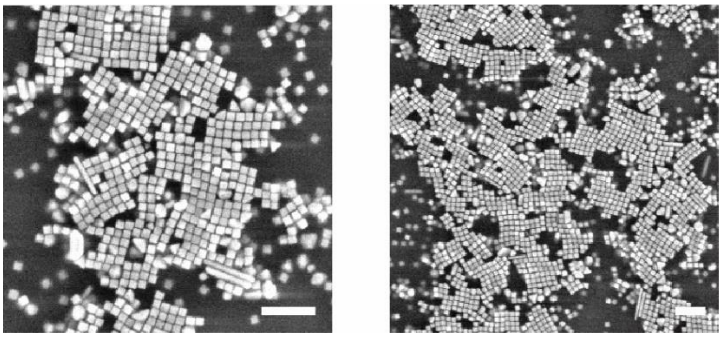

Supplementary Figure 19. SEM images of AgNCs functionalized with 50 µM PEG20k and 6 µM C_{16} in feedstock assembled by gravitational sedimentation and dropcast on Si substrate (scale bar 500 nm). The NCs assembled into high-density, closed-packed 3D superstructures, which are different as Janus-type structures, where the nanocubes are selectively functionalized on only a few facets typically results in 1D (2 facets) or 2D (4 facets) superstructures, and are indicative of surface to be homogeneous.[5]

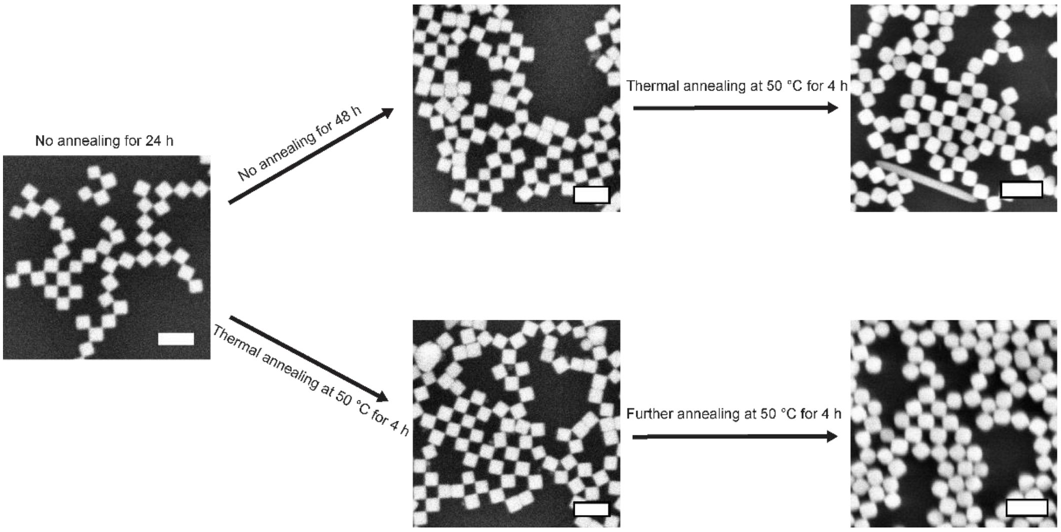

Supplementary Figure 20. Thermal annealing of the Langmuir–Blodgett (LB) films under mild conditions (50 °C, 4 hours, scale bar 100 nm). Longer thermal annealing times or increased temperatures leads to a shape change, where the nanocubes become rounded and approach a spherical shape. We observe that mild thermal annealing accelerates nanocube assembly, which normally takes up to 72 hours at room temperature. However, thermal annealing does not promote the assembly of larger scale, extended checkerboard lattices.

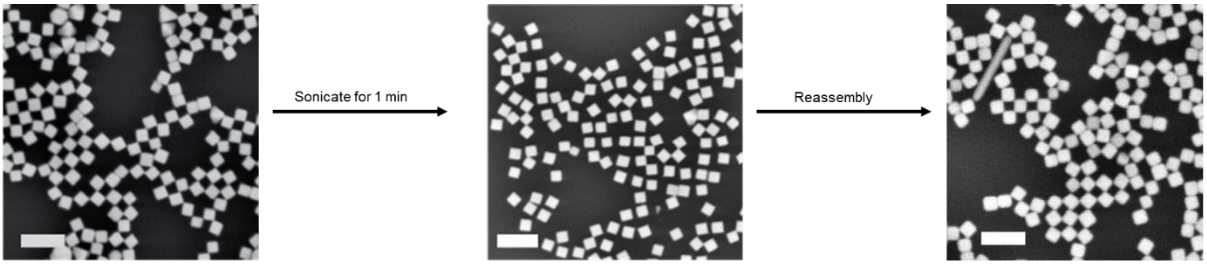

Supplementary Figure 21. Agitation annealing by sonicating the assembled checkerboard film to disassemble NCs and reassembly (scale bar 500 nm). The redistributed NCs reassemble into checkerboard lattices after 24 hours without a significant difference in the quality or size of the checkerboard domains.

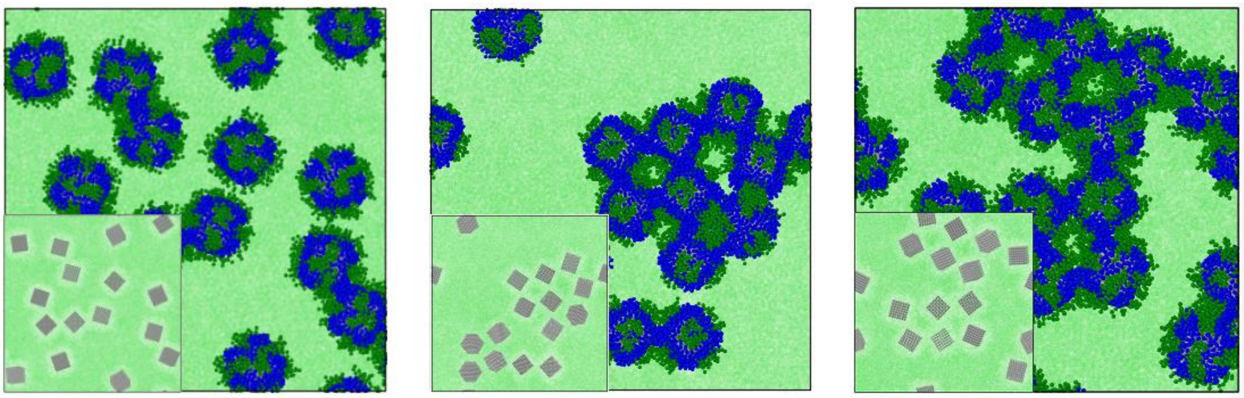

Supplementary Figure 22. Three independent simulations of the assembly of 16 grafted nanocubes at $\boldsymbol{\Gamma_{\mathrm{g}} = 1.0}$ chains $\boldsymbol{\sigma^{-2}}$ and number percentage of hydrophobic ligands = 67%.

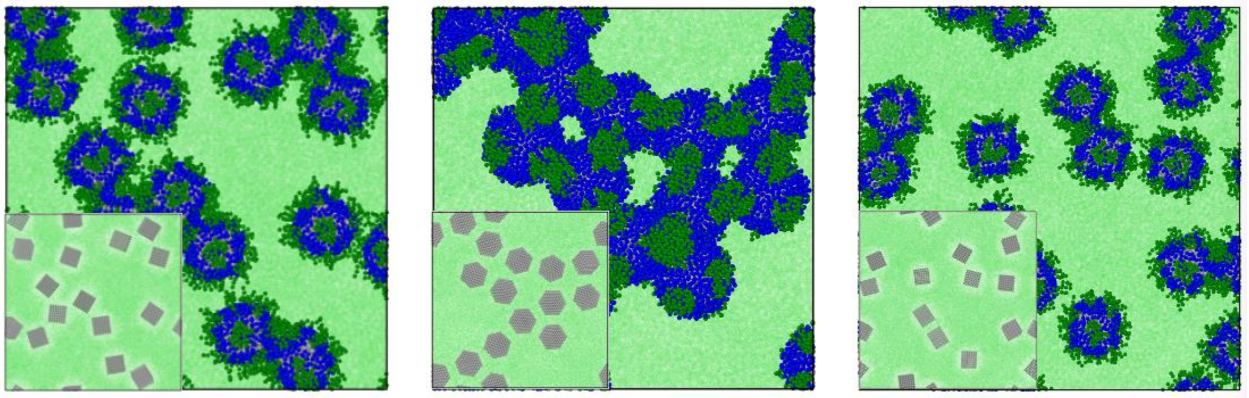

Supplementary Figure 23. Three independent simulations of the assembly of 16 grafted nanocubes at $\boldsymbol{\Gamma_{\mathrm{g}} = 0.75}$ chains $\boldsymbol{\sigma^{-2}}$ and number percentage of hydrophobic ligands = 67%.

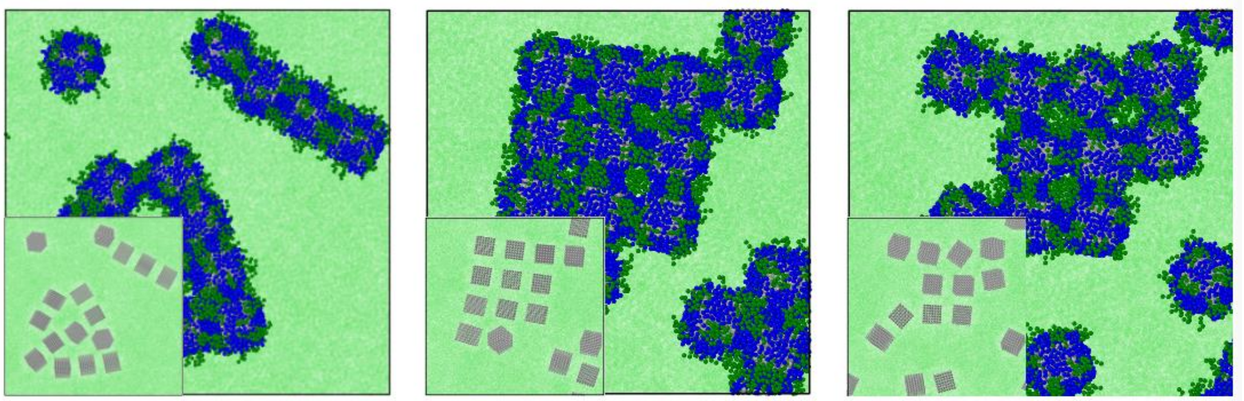

Supplementary Figure 24. Three independent simulations of the assembly of 16 grafted nanocubes at $\boldsymbol{\Gamma_{\mathrm{g}} = 0.75}$ chains $\boldsymbol{\sigma^{-2}}$ and number percentage of hydrophobic ligands = 75%.

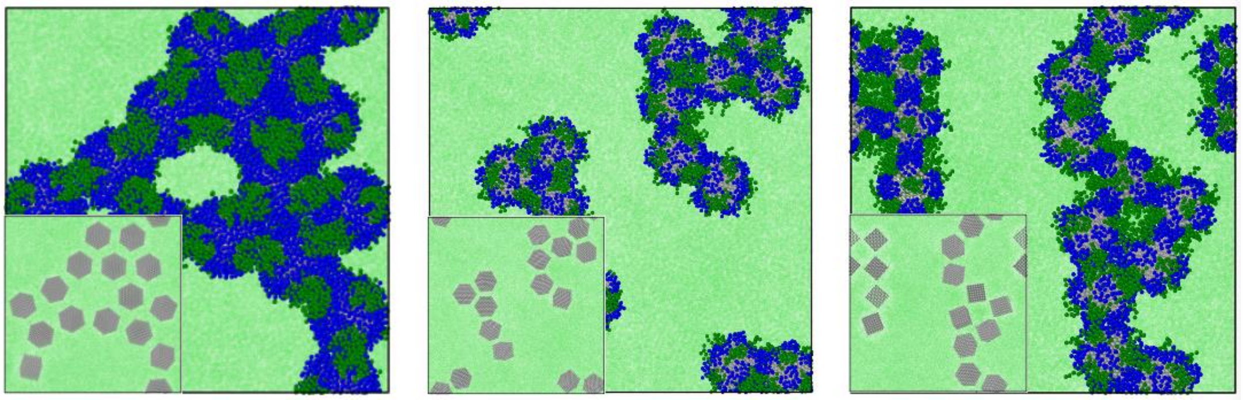

Supplementary Figure 25. Three independent simulations of the assembly of 16 grafted nanocubes at $\boldsymbol{\Gamma_{\mathrm{g}} = 0.50}$ chains $\boldsymbol{\sigma^{-2}}$ and number percentage of hydrophobic ligands = 67%.

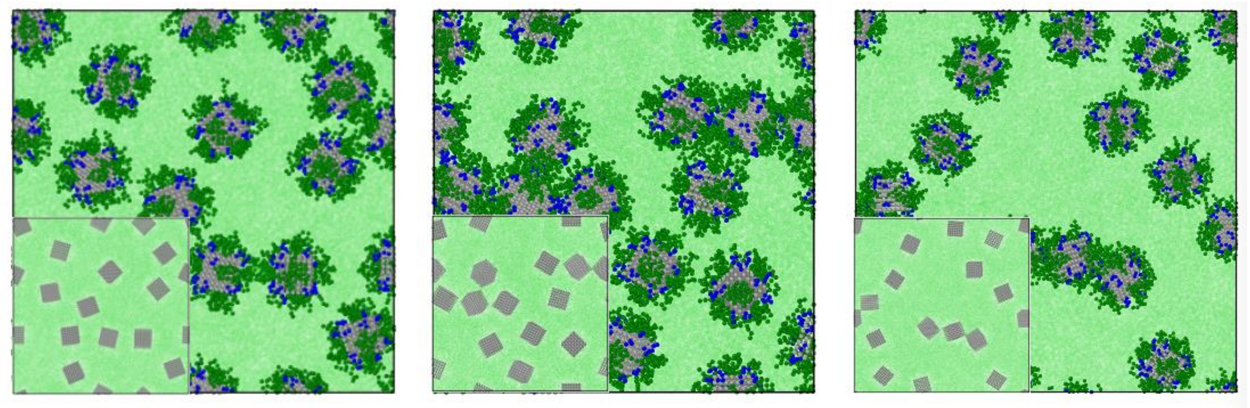

Supplementary Figure 26. Three independent simulations of the assembly of 16 grafted nanocubes at $\boldsymbol{\Gamma_{g} = 0.25}$ chains $\boldsymbol{\sigma^{-2}}$ and number percentage of hydrophobic ligands = 25%.

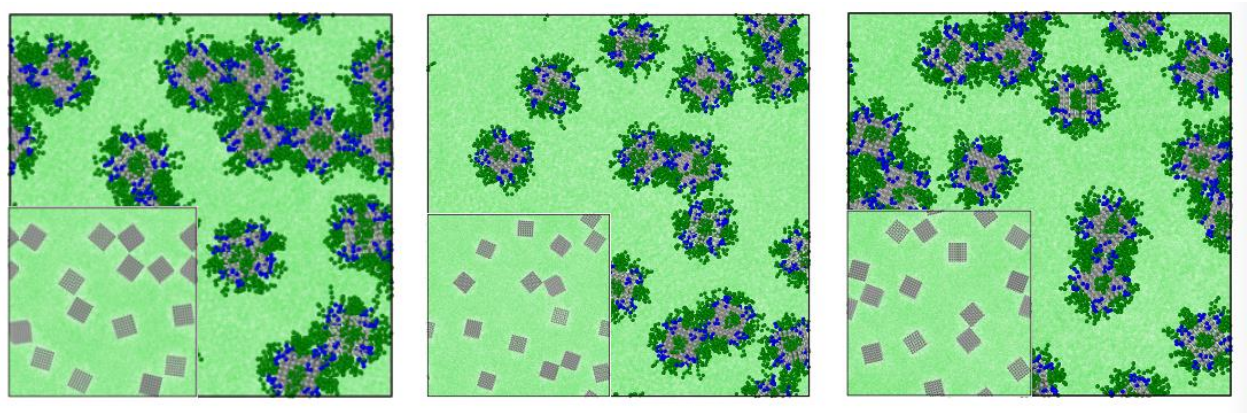

Supplementary Figure 27. Three independent simulations of the assembly of 16 grafted nanocubes at $\boldsymbol{\Gamma_{g} = 0.25}$ chains $\boldsymbol{\sigma^{-2}}$ and number percentage of hydrophobic ligands = 50%.

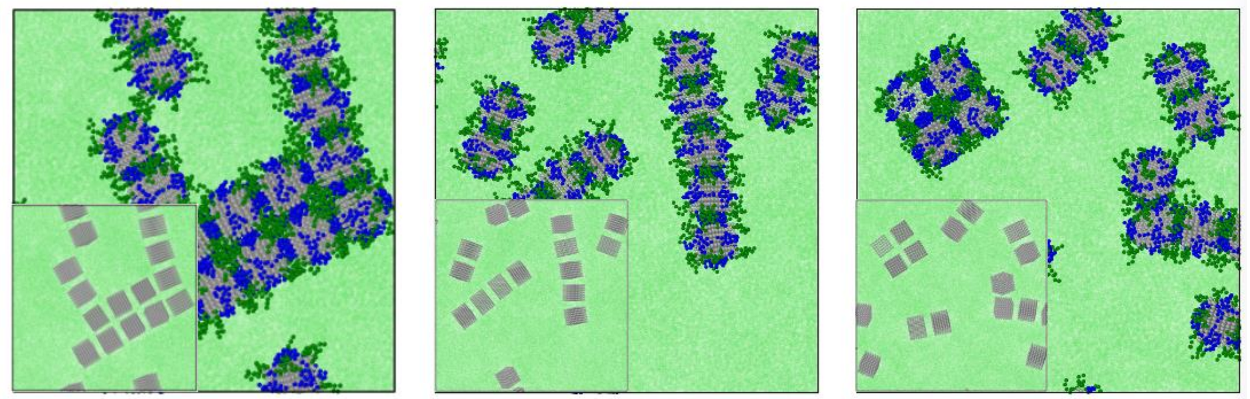

Supplementary Figure 28. Three independent simulations of the assembly of 16 grafted nanocubes at $\boldsymbol{\Gamma_{g} = 0.25}$ chains $\boldsymbol{\sigma^{-2}}$ and number percentage of hydrophobic ligands = 67%.

Supplementary Discussion 1

Simulation results of the dispersed AgNC film (Supplementary Figure 5b), where AgNCs are modelled with a spacing of > 100 nm to approximate little-to-no plasmonic coupling, shows a dip in the reflection spectra that redshifts from λ=410 nm to λ=459 nm as the height of the air-water interface increases from the bottom facet of the AgNC (h=0 nm) to the top facet of the AgNC (h=80 nm), respectively. This is attributed to peak splitting of the quadrupolar localized

surface plasmon resonance (LSPR) mode of an isolated AgNC due to anisotropic dielectric environment generated by partially submersion of the nanocube in water. Dispersed AgNC films were experimentally obtained using as-made AgNCs that are capped with PVP. The measured reflection spectra of these AgNCs (Supplementary Figure 5c) when dispersed on the air-water interface show a reflection dip at λ = 442 nm, consistent with the simulation results for h = 70 (λ = 451 nm), indicating that 89% of the AgNC volume is submerged below the air-water interface. This is also consistent with CG-MD simulation results in Figure 2 of the main manuscript. This air-water interface height of h = 70 was then used to simulate the reflectance of checkerboard and edge-edge AgNCs with a 3.5 nm interparticle gap. The simulated spectrum shows a reflection dip at λ = 660 nm (well beyond what is observed for dispersed AgNCs) that is attributed to quadrupole-quadrupole LSPR coupling (Supplementary Figure 5d). This is consistent with a large absorption peak also observed in FDTD simulations. The simulated reflection spectrum also exhibits a reflection peak shoulder at λ = 516 nm which is attributed to weaker plasmonic coupling between higher order modes. Both features match well with the experimental data obtained for edge-edge assembled AgNCs. These edge-edge AgNCs films were obtained with the same surface chemistries described in the main manuscript (50 µM PEG20k-SH and 6 µM C_{16}-SH), but with much lower AgNC film densities and with large variances in edge-edge AgNC connection geometries when compared to the FDTD models. These results strongly indicate that the optical properties of the macroscopic (≈8.6 mm^2 area) AgNC assembly is dominated by plasmonic coupling that results directly from edge-edge interactions.

Supplementary References

1 Wochner, P. *et al.* X-ray cross correlation analysis uncovers hidden local symmetries in disordered matter. *Proc. Natl. Acad. Sci.* **106**, 11511-11514 (2009).

2 Altarelli, M., Kurta, R. & Vartanyants, I. X-ray cross-correlation analysis and local symmetries of disordered systems: General theory. *Phys. Rev. B* **82**, 104207 (2010).

3 Kurta, R., Altarelli, M., Weckert, E. & Vartanyants, I. X-ray cross-correlation analysis applied to disordered two-dimensional systems. *Phys. Rev. B* **85**, 184204 (2012).

4 Palik, E. D. *Handbook of Optical Constants of Solids*. (Academic Press, 1985).

5 Rycenga, M., McLellan, J. M. & Xia, Y. Controlling the Assembly of Silver Nanocubes through Selective Functionalization of Their Faces. *Adv. Mater.* **20**, 2416-2420 (2008).
